# Editorial: Multimodal behavior from animals to bio-inspired robots

**DOI:** 10.3389/fnbot.2023.1160549

**Published:** 2023-02-21

**Authors:** Yaguang Zhu, Poramate Manoonpong, Qiao Hu

**Affiliations:** ^1^State Key Laboratory of Robotics and Systems (HIT), Harbin, China; ^2^The Key Laboratory of Road Construction Technology and Equipment of MOE, Chang'an University, Xi'an, China; ^3^Bio-Inspired Robotics and Neural Engineering Laboratory, School of Information Science and Technology, Vidyasirimedhi Institute of Science and Technology, Rayong, Thailand; ^4^Embodied AI and Neurorobotics Laboratory, SDU Biorobotics, The Mærsk Mc-Kinney Møller Institute, University of Southern Denmark, Odense, Denmark; ^5^School of Mechanical Engineering, Xi'an Jiaotong University, Xi'an, China

**Keywords:** multimodal behavior, bio-inspired robot, locomotion control, embodied intelligence, multimodal sensory integration

## Introduction

Animals exhibit multimodal behavior, which means that they can behave in different motion patterns, such as crawling, running, jumping, hunting, and escaping. Quadrupeds can maintain balance and coordination in challenging terrains. Amphibians can transit on land and in water. Insects can freely fly and crawl. These innate locomotion talents are simple and natural to animals and enable them to survive in nature by efficiently exploiting their body morphology and neural control system. Thus, it is very important to investigate the mechanism and influencing factors of multimodal behavior, especially for the development of dexterous, agile, and stable robots through biologically inspired guidance.

Various attempts, including the use of hybrid structure systems, neural control, and embodied intelligence, have been made to mimic such impressive multimodal behaviors for robots, e.g., the turtle robot (Baines et al., 2022), salamander robot (Ijspeert et al., 2007), gecko robot (Shao et al., 2022), quadruped robot (Miki et al., 2022), millipede robot (Homchanthanakul and Manoonpong, 2023), and dung beetle robot (Xiong and Manoonpong, 2021). However, in comparison to their biological counterparts, robots still fail in terms of adaptability, flexibility, and versatility, because multimodal behaviors involve the synthesis of structure, control, planning, and optimization. Although the multimodal concept, previously realized in vehicle engineering and electrical engineering, will greatly improve the agility and intelligence of bio-inspired robots' behavioral control, it also poses new challenges. For example, the muscle tissue and body morphology of an animal are quite different from the actuators of robots, and the animal's neural system is also much more complex than the control system of robots. To bridge the gap between biology and bio-inspired robots, these factors must be taken into account (Manoonpong et al., 2021). Therefore, further research and discussions relating to the multimodal concept of robot behavior still need to be conducted.

The goal of this Research Topic is to demonstrate the recent progress in relation to “*Multimodal Behavior from Animals to Bio-Inspired Robots*,” including the design of the mechanical structure, sensory systems, control strategy, and enhancements between biology and engineering. The topic contains four articles, each addressing the control methods for the multimodal behavior of bionic robots with different structures, including biped and hexapod robots, as well as sensor systems and reinforcement learning methods for multimodal behavior.

## Multimodal behavior

Researchers have developed many bionic robots, with the configuration and locomotion mostly inspired by animals, e.g., the typical gaits (walking and running) of quadruped robots (Lee et al., 2020), flying robots with complex wing dynamics (Ramezani et al., 2017), and a swimming robot undulating its body and/or flapping its fins (Ijspeert, 2014). These behaviors not only bring a unique perspective to research on biology and bionic robotics but also pose substantial technological challenges for robot modeling, design, and control.

### Structure and body morphology

For bionic robots, different structures and morphology should be developed and designed according to the application scenarios. Thus, the topics of concern and unsolved problems will also vary accordingly. In this Research Topic, Zhao et al. focus on exploring the effects of the serial and parallel elasticity on hopping with a two-segmented robotic leg called an electric-pneumatic actuation (EPA)-Hopper. The performance of the serial and parallel pneumatic artificial muscles (PAM) is evaluated with respect to four criteria: efficiency, performance, stability, and hopping robustness against perturbations. The serial PAM has a more pronounced impact than the parallel PAM on these criteria and can help to further understand the human hopping mechanism and support the design and control of legged robots and assistive devices. Strohmer et al. developed a spiking neural network capable of continuously changing amplitude, frequency, and phase online. The network is deployed to observe the meaningful role of network topology in locomotion on the Modular Robot Framework (MORF), which is both a simulated robot in V-REP and a physical hexapod robot with 18 degrees of freedom. The neural control of locomotion relates back to biology where it can provide theoretical evidence that is not currently testable on behaving insects.

### Behavioral control strategy

The high dynamics control strategy of the bio-inspired robot is impressive and has a history of technology, such as a non-linear program (NLP) (Jenelten et al., 2022), Linear Quadratic Regulator (LQR) (Klemm et al., 2020), and Model Predictive Control (Di Carlo et al., 2018). Besides model-based control, robots driven by neural control architectures, that can respond to internal and external information and implement various behaviors, have superior upper-level capabilities in terms of behavior pattern diversity with smooth transition (Zhu et al., 2022) as well as versatile locomotion on complex terrains (Picardi et al., 2020; Thor and Manoonpong, 2022). In this Research Topic, Bonzon defines a neural basis for such behaviors, potentially learned by bio-inspired artifacts. The stochastic decision tree that drives this behavior is then transformed into a plastic neuronal circuit. The principle of using synchronized multimodal perceptions in association with the Hebb principle of wiring together neuronal cells is induced. The emergence of behaviors allows for a strict delineation of successive complexity levels. The isolation of levels allows for simulating yet unknown processes of cognition independently of their underlying neurological grounding. Koseki et al. provide a framework that reproduces multimodal bipedal locomotion using passive dynamics through deep reinforcement learning (DRL). By carefully planning the weight parameter settings of the DRL reward function during the learning process based on a curriculum learning method, the bipedal model successfully learned to perform multimodal behaviors (walk, run, and gait transitions). These results indicate that DRL can be applied to generate various gaits with the effective use of passive dynamics.

## Concluding remarks

The Research Topic presents multimodal behavior approaches of bio-inspired robots, considering mechanical design, sensory integration systems, control strategies, and applications in different environments. The studies on this topic cover multimodal behaviors for the hopping robot, biped robot, and hexapod robot. The results from these studies confirm that the sensory system provides support for the decision-making of multimodal behaviors. Furthermore, the neural control architecture can easily realize a variety of gait patterns, while the reinforcement learning and control methods ensure the stability and reliability of the related locomotion. Given these and further studies, robots with delicate structures and systems are likely to have more complex multimodal behavior and can be more similar to the behavior mechanisms and physiological functions of animals in the future.

## Author contributions

All authors listed have made a substantial, direct, and intellectual contribution to the work and approved it for publication.
